# Conservative management of post traumatic thoraco diaphragmatico biliary fistula

**DOI:** 10.4103/0970-2113.71962

**Published:** 2010

**Authors:** N. C. Kajal, Babu Lal, Sandeep Gupta, Ramesh Attri, Onkar Gupta, Nadia Kajal

**Affiliations:** *Department of Chest and TB-Govt. Medical College, Amritsar, Punjab, India*

**Keywords:** Aneurysm, dissecting, effusion

## Abstract

We report herein a case of thoraco diaphragmatico biliary fistula in a 24-year-old male who was managed conservatively with antibiotics and tube thoracostomy and had complete radiological clearance.

## INTRODUCTION

Thoraco diaphragmatico biliary fistula is a rare manifestation of post traumatic biliary disruptions. Given their rarity, it is not surprising that there is little consensus on the optimal management of these fistulas.

## CASE REPORT

**Figure 1:**
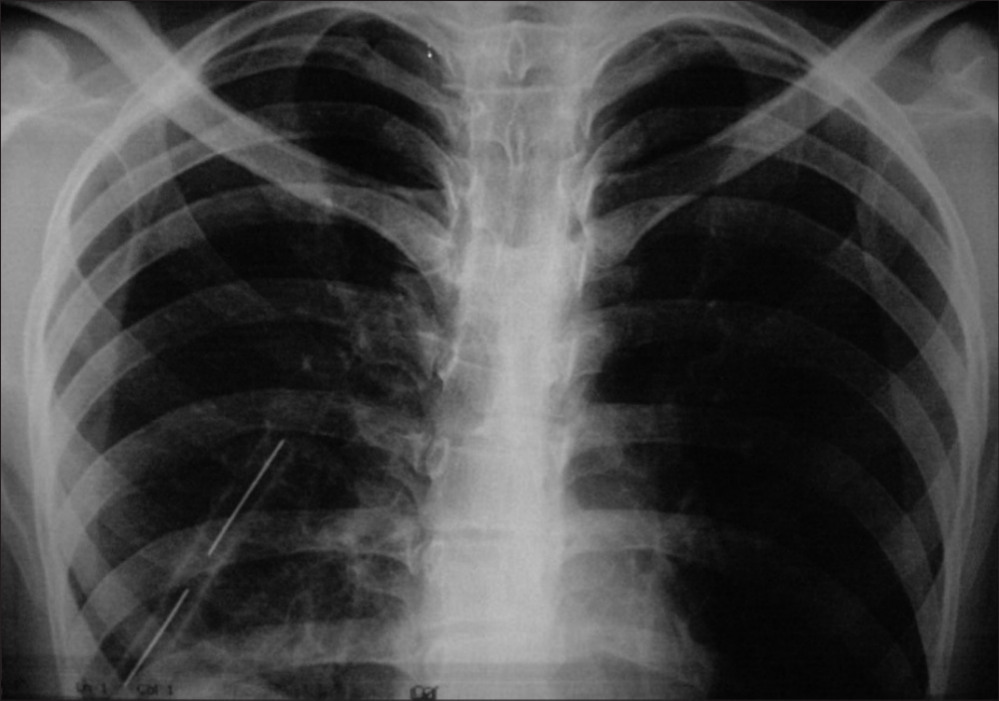
Tube thoracostomy

**Figure 2:**
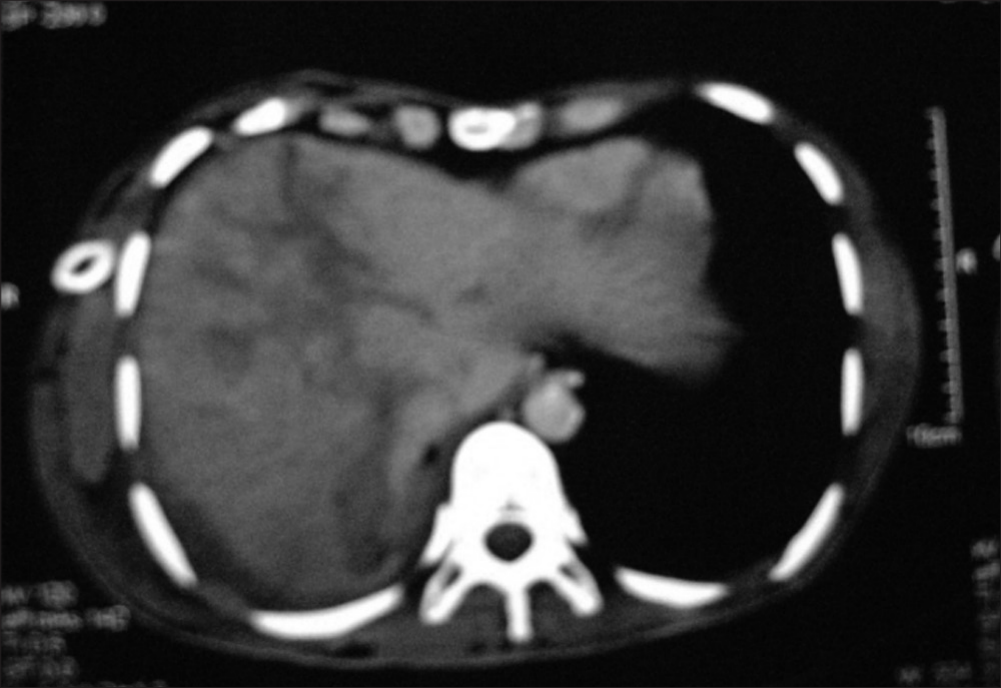
Diaphragmatic tear

A 24-year-oldmale patient with an alleged history of RSA presented to our hospital with a penetrating wound, with an iron rod, on the right side of anterior chest wall. After getting first aid, he was referred to our hospital for further management. On examination, patient had decreased breath sounds on right side. CXR PA view showed homogenous opacity on right side with rising level of fluid and blunting of CP angle. Thoracocentesis was done and it revealed hemothorax. Tube thoracostomy was performed and blood was drained [Figure 1]. Later contrast enhanced tomographic (CECT) scan of chest and abdomen was done which showed right side surgical emphysema with fracture of right 12^th^ rib. Right lung showed contusion in the lower lobe with moderate hemothorax with basal atelectasis with minimal pneumothorax. The visualized right lobe of liver showed large lacerations with breach in the superior surface of liver below the right diaphragm [Figure 2]. After two days of thoracostomy, when the blood was drained, brownish colored fluid appeared in the bag. Thus, the diagnosis of thoraco diaphragmatico biliay fistula was made. Initially, broncho pleural fistula was patent but it got closed after 25 days of thoracostomy and bile also decreased in amount to approximately 50 ml/day.

## DISCUSSION

Early diagnosis of TBF is crucial in the management of this condition. A delayed diagnosis leads to the development of several complications that may warrant extensive surgery.[1] Bile has been shown to have a corrosive effect upon the lung and pleural space.[2,3] A high index of suspicion in the appropriate clinical situation is therefore mandatory. The presence of bile on thoracentesis of a pleural effusion and bilioptysis are pathognomonic for TBF. Bilioptysis may range in presentation from bile-stained sputum to the expectoration of large volumes of bile occasionally approaching a liter.[4] Pleurobiliary fistula may predispose to a loculated bilious empyema; the consequent development of pleural adhesions may entrap the lung, thereby compromising lung function.[5] Bronchobiliary fistula may predispose to necrotizing bronchitis or bronchopneumonia; rarely a chronic indirect pneumonitis may develop.[4]

The initial management of patients presenting with thoraco diaphragmatico biliary fistula is conservative management with tube thoracostomy or drainage of sepsis when appropriate, or both; antibiotics are routinely administered.

Endoscopic cholangiography may demonstrate the fistulous tract and identify distal biliary obstruction, which is crucial for the persistence of TBF. Furthermore, endoscopic sphincterotomy may be undertaken during this study.

Endoscopic retrograde cholangiography is advised if symptoms persist to delineate the thoracobiliary communications and undertake sphinteroplasty.

## CONCLUSION

Thoraco diaphragmatico biliary fistulas can be successfully managed using a conservative approach. Surgery should be reserved for persistence of symptoms after exhaustion of this approach.
